# Nutritional value of black soldier fly (*Hermetia illucens*) larvae processed by different methods

**DOI:** 10.1371/journal.pone.0263924

**Published:** 2022-02-25

**Authors:** Nor Fatin Najihah Mohamad Zulkifli, Annita Yong Seok-Kian, Lim Leong Seng, Saleem Mustafa, Yang-Su Kim, Rossita Shapawi

**Affiliations:** Borneo Marine Research Institute, Universiti Malaysia Sabah, Jalan UMS, Kota Kinabalu, Sabah, Malaysia; Universita degli Studi della Basilicata, ITALY

## Abstract

Nutritional value of black soldier fly (*Hermetia illucens*) larvae (BSFL) processed by three different methods of treatment was compared. The resulting products were the spray-dried BSFL (SPR), oven-dried BSFL 1 (OVN1) and oven-dried BSFL 2 (OVN2). Proximate chemical composition, and profiles of amino acids, fatty acids, minerals, heavy metals, vitamins and nucleotides were analysed and compared. The tested BSFL meals were considered to have a good profile of essential amino acids (EAAs), with leucine, lysine, valine, and histidine being the dominant EAAs. Their content of saturated fatty acids exceeded that of the unsaturated fatty acids. Vitamins B1, B2, and C were also present in the samples. Minerals such as calcium, potassium, phosphorus, sodium, magnesium, zinc, iron, manganese and copper were found to be in adequate amounts in almost all the samples. Heavy metals in the BSFL meals were mostly below 1g kg^-1^. Nucleotides such as inosine monophosphate and uridine monophosphate occurred in all the BSFL meals. Other nucleotides, including guanosine monophosphate, adenosine monophosphate, xanthosine monophosphate, and cytidine monophosphate were detected in either or both of SPR and OVN2. In general, the nutritional value of the BSFL meals tested in the present study was influenced by the method of processing.

## Introduction

Feeding farmed fish is a major challenge for development of aquaculture. Use of bulk quantities of wild fish from the ocean is one of the main concerns expressed about aquaculture. Sustainably increasing production through scientific and technological advances in finfish feeds is considered a necessary pathway to increasing the contribution of this sector to food security [1]. Aquafeeds that are nutritious and acceptable to the cultured fish should also comprise ingredients that are economically viable and are sourced from environmentally sustainable resources. There is an increasing number of investigations showing the importance of insect meal in enriching dietary intake of protein, polyunsaturated fatty acids and several other nutrients that play vital roles in growth and wellbeing of the aquaculture fish [2]. These qualities make the insect meal a suitable alternative to fish meal and oil.

Black soldier fly larvae (BSFL, *Hermetia illucens*) have emerged as among the more environmentally sustainable choice for aquafeeds. BSFL meal contains a high level of protein, with the amino acid profile similar to fish meal and other nutrients that make it a well-balanced feed [3, 4]. *H*. *illucens* is widely distributed in warm and temperate regions and is a potential protein source. It has a short life cycle and is easy to breed and grow. Also, it does not require feeding at certain stages of its life cycle. BSFL can be reared on a wide variety of organic waste materials and thus, it provides a potential approach to reducing the volume of this waste [5]. Due to their small size, these insects require less space if they were to be bred and farmed. It was also reported that the ammonia emissions associated with insect rearing are also much lower than the domestic livestock [6]. In a study conducted by Oonincx *et al*. [7], insects were reported to emit 80 times less ammonia compared to cattle on a weight for weight basis. This is significant because methane has 25 times impact on global temperature than carbon dioxide. Wang and Shelomi [8] have also highlighted this advantage with specific reference to BSFL. Apart from that, BSFL has also been bio-prospected for its potential as a source of antimicrobial peptides [9–12], chitin [13–15] and lipids [16].

In general, protein sources should meet certain conditions for their production, such as regular availability in quantity, economic value, non-competition with human resources and environmental sustainability. BSF seems to fit these criteria. At present, the BSFL meal is mainly utilized for livestock feed production rather than for human consumption [17, 18]. The animal feed industry world-wide is seeking alternative protein from sustainable sources. In addition to protein, BSFL also contains fatty acids and polysaccharides and possibly other substances of nutritional value. Currently, the use of BSFL is receiving more attention in aquaculture feed industry as an effort to reduce dependency on fish meal for protein and oil.

A comprehensive biochemical analysis of feed stuffs is important for assessment of their nutritional value and sharing the information with companies and consumers. Studies on insect body composition show large differences between different species. In addition, the nutritional composition of insects can be significantly influenced by methods of their farming and processing for feed development [19]. Therefore, it is beneficial to investigate the nutritional composition of three different types of BSFL meals available in the local market. Fruit and vegetable wastes as substrates for rearing BSFL are widely used because of higher levels of long-term omega-3 fatty acids, EPA (eicosapentaenoic acid) and DHA (docosahexaenoic acid) that these products have been reported to achieve in the insect compared to other substrates sourced from terrestrial plants [20], and their bulk availability. In the present study, the nutritive values of three different types of BSFL were investigated. This enabled us to undertake focused studies for generation of comprehensive data through experimental trials. Proximate composition, and concentrations of amino acids, fatty acids, vitamins, minerals, nucleoside and nucleotide contents of the larvae meal were determined to understand if these chemical profiles are comparable to fish-based diets and will be able to support growth and health of the fed fish in culture systems.

## Materials and methods

### BSFL processing

Three different types of BSFL were used in this investigation. These included spray-dried BSFL (SPR), oven-dried BSFL 1 (OVN1) and oven-dried BSFL 2 (OVN2). Both SPR and OVN1 were fed organic agroindustry by-products. BSFL were dried in an oven at 50°C for 24–36 hours until it is fully dried prior for grinding process. After that, the larvae were grinded into powder form using a blender. This physical form has been referred to as the BSFL meal in the manuscript. Lastly, the BSFL meal was sieved using a sieve to obtain homogenized powder and then tightly packed and stored at -5°C before being used for analysis. SPR and OVN1 meal were obtained from a BSF pilot farm located in Kuala Lumpur, Malaysia. Both SPR and OVN1 used BSFL fed with agroindustry by-products. SPR was dried by the help of spray-drying technique and BSFL was dried using an oven-drying method. Meanwhile OVN2 meal was obtained from an individual insect breeder located in Likas, Sabah, Malaysia. They were fed with food waste and then turned into BSFL powder in the laboratory using an oven-drying method at temperature of 60°C in an overnight treatment.

### Chemical analysis

#### Proximate analysis

Crude protein was determined by the Kjeldahl method using an automatic system (Kjeltec 2300). Crude lipid was gravimetrically measured by the ether-extraction method in a soxhlet extraction unit (Soxtec 2043). Crude ash content was determined as the residue remaining after incineration of samples at 550°C in a muffle furnace for 6 hours. Moisture was quantified by thermogravimetric method using an oven by drying samples at 105°C until constant weight [21].

#### Fatty acid analysis

For hydrolysis of lipids, the sample was refluxed with 1M solution of potassium hydroxide in 95% ethanol before being extracted with hexane-diethyl ether. The solvent-extracted samples were then subjected to centrifugation at 3,000 rpm for 10 minutes before being washed with water. Subsequently, the contents were dried by evaporation process in a rotary evaporator to obtain the free fatty acids. The fatty acid methyl esters (FAMEs) were prepared with methanolic sulphuric acid. The fat was dissolved in toluene and 1% sulphuric acid in methanol before storage overnight. After that, water containing 5% sodium chloride was added. The FAMEs were then washed with 2% potassium carbonate and then dried by passing through a short column of anhydrous sodium sulphate. FAMEs were then diluted with hexane before injection into a Gas Chromatography column.

#### Vitamin analysis

Vitamins A, B1 and B2 were analysed using a High-Performance Liquid Chromatography detector (HPLC, Waters Alliance, model E2695 PDA), while Vitamin C was determined by HPLC (Agilent model 1200 Series DAD) detector. The sample was subjected to grinding before being extracted for carotenoids using tetrahydrofuran that contained butylated hydroxytoluene (BHT) in the presence of sodium sulphate and calcium carbonate for a few times followed by HPLC processing.

Vitamins B1 (Thiamine) and B2 (Riboflavin) were extracted from the sample by acid hydrolysis followed by enzymatic hydrolysis. The aqueous extract was injected onto a reverse phase HPLC column. The fluorescence of riboflavin was measured, and the thiamine was determined after post-column derivatisation with alkaline potassium ferricyanide that converts the thiamine into thiochrome.

For vitamin C (ascorbic acid), the sample was homogenised in 3% metaphosphoric acid. The homogenate was filtered, and a sample of the extract was chromatographed on RP C18 column by means of HPLC. Evaluation was then performed by differentiating the peak area against the ascorbic acid standard.

#### Phosphorus analysis

The phosphorus content of the sample was analysed using the Inductively Coupled Plasma—Optical Emission (ICP-OES). For digestion of the sample, the organic matter was removed using concentrated nitric acid as the oxidising agent. Concentrated hydrochloric acid was then added before the sample was digested in a block digester until a clear solution was obtained. The solution so prepared was nebulised into argon plasma, where all the components were vaporized. The phosphorus element was then atomized and excited, and the emitted radiations were measured at various wavelengths simultaneously.

#### Amino acid analysis

The samples were subjected to hydrolysis using hydrochloric acid under total hydrolysis condition. A measured small amount of the amino acid solution was then derivatized with AccQ-Fluor Reagent together with aminobutyric acid as the internal standard. The amino acid derivatives were analysed using HPLC (Waters–Alliance e2695) with Fluorescence detector (2475-waters). The amount of amino acid (ng) was determined by external standard calibration with aminobutyric acid (AABA) to compensate for variation in derivatization between the samples.

#### Mineral and heavy metal analysis

The mineral contents of the BSFL were analysed using an ICP-OES (Perkin Elmer Optima 5300 DV ICP-OES, USA). Before the analysis, the BSFL was digested using nitric acid and hydrochloric acid. Dried sample of 0.1 g was weighed and transferred into a dry digestion flask. After that, 5 ml of nitric acid (69% concentration) was added into the flask and the contents were heated on a heating mantle in a fume hood until the mixture started to boil and white precipitate was obtained. The flask was then cooled to room temperature, and this was followed by addition of 3 ml of hydrochloric acid and heating until green colour of the sample turned yellow, orange or red. After the colour was changed, the flask was heated again for 10 minutes to prevent the colour reversal. The sample was then cooled to room temperature and poured into a 100 ml volumetric flask. Distilled water was added until 100 ml volume and the contents were allowed to stand until analysis in an ICP-OES.

#### Nucleotide analysis

The contents of nucleotides in the three BSFL and the BSFL diets were determined using the UPLC-TQ-MS/MS method. Dried sample powder measuring 1 g was sonicated with 50mL distilled water at room temperature and extracted by centrifugation at 13,000 rpm for 10 min. The supernatant was then kept at 4°C and filtered using a 0.22 μm membrane filter prior to the injection. Individual standards were also prepared by dissolving in 10mL of distilled water, kept at 4°C and filtered using a 0.22 μm membrane filter before injection. Subsequently, the chromatographic analysis was accomplished using Waters Acquity UPLC system (Waters, Milford, MA, USA) equipped with an Acquity UPLC HSS T3 c18 column (1.8 μm, 2.1 mm x 150 mm). The column temperature was maintained at 35°C. The flow rate of the mobile phase was 0.3 mL/min. and the injection volume was 1 μL.

#### Statistical analysis

Data on proximate composition, and concentration of minerals and heavy metals were subjected to one-way analysis of variance (ANOVA) and Duncan’s post-hoc test for determining the significance of difference at p<0.05 using Statistical Packages of Social Sciences Version 20. Due to limited sample amount, data on amino acids, fatty acids and vitamins were based on single analysis, thus no statistical analysis was performed.

## Results

### Proximate composition of the BSFL

Table 1 shows the proximate composition of the three differently processed BSFL: SPR, OVN1 and OVN2. The moisture, ash, protein, lipid, fibre, and NFE contents of the BSFL ranged from 3.21–7.10%, 7.26–8.27%, 39.38–48.20%, 25.69–38.36%, 7.41%-9.96% and 6.45–7.88%, respectively. Except for NFE, there were significant differences in the proximate composition of the different types of BSFL.

**Table 1:** Proximate composition of the BSFL (% DM).

Proximate analysis		BSFL	
	SPR	OVN1	OVN2
Crude protein	48.20 ± 0.05^c^	47.46 ± 0.14^b^	39.38 ± 0.16^a^
Crude lipid	25.69 ± 0.12^a^	28.43 ± 0.05^b^	38.36 ± 0.19^c^
Crude fibre	9.96 ± 0.70^b^	9.48 ± 0.04^b^	7.41 ± 0.03^a^
Moisture	7.10 ± 0.05^c^	3.21 ± 0.03^a^	5.32 ± 0.03^b^
Ash	8.27 ± 0.07^b^	8.19 ± 0.05^b^	7.26 ± 0.03^a^
NFE^1^	7.88 ± 0.71	6.45 ± 0.09	7.59 ± 0.24

^1^NFE (Nitrogen-free extract) = 100 –(Crude protein + crude lipid + crude fibre + ash).

Values are mean ± SE (n = 3).

#### Amino acid composition

The amino acid composition of the three BSFL is presented in Table 2. In all BSFL samples, leucine, lysine, valine, and histidine were the most abundant essential amino acids (EAA) observed, whereas aspartic and glutamic acids were the most abundant non-essential amino acids. SPR was observed to contain the highest values of most of the amino acids studied compared to the OVN1 and OVN2 samples.

**Table 2:** Amino acid composition of the BSFL (% DM).

Amino acid	BSFL		
	SPR	OVN1	OVN2
Histidine	2.77	2.70	2.08
Threonine	1.94	1.85	1.42
Arginine	2.55	2.25	1.80
Methionine	1.07	0.93	0.61
Valine	3.09	2.92	2.29
Phenylalanine	2.11	1.98	1.35
Isoleucine	2.40	2.28	1.76
Leucine	3.62	3.51	2.67
Lysine	3.60	2.87	2.44
Aspartic acid	5.09	4.55	3.30
Cysteine	0.16	0.15	0.12
Glutamic acid	6.05	5.63	4.59
Serine	2.06	1.88	1.55
Glycine	0.25	0.22	0.12
Alanine	3.06	3.00	3.13
Proline	2.86	2.81	2.30
Hydroxyproline	0.03	0.03	0.03
Tyrosine	3.09	2.60	1.71

#### Fatty acid composition

Fatty acid composition of the BSFL preparations (Table 3) obtained in this study shows that OVN1 and OVN2 BSFL have high concentration of saturated fatty acid (SFA) compared to the unsaturated fatty acid; monounsaturated fatty acid (MUFA) and polyunsaturated fatty acid (PUFA). Greater concentration of SFA such as lauric acid (C12:0) and palmitic acid (C16:0) was observed in every BSFL. SPR had the lowest concentration of SFA among OVN1 and OVN2. Concentration of MUFA in the SPR BSFL exceeded that in the SFA. Among the three BSFL, SPR also had the highest concentration of omega-3 and omega-6. However, the ratio of omega-3 to omega-6 was similar in SPR and OVN1 while it was higher in OVN2 where the concentration of omega-3 in all BSFL studied was lower than the amount of omega-6.

**Table 3:** Fatty acid composition (% of total fat) of the BSFL.

Fatty acid (%)	BSFL		
	SPR	OVN1	OVN2
10:0	ND	0.85	0.44
12:0	17.89	37.18	36.59
14:0	5.21	8.09	11.77
16:0	20.65	24.59	24.00
18:0	2.95	3.32	4.42
21:0	ND	0.82	ND
22:0	ND	ND	0.26
Sum SFA	46.69	74.83	77.47
16:1	1.75	2.67	2.55
18:1- cis n9	9.28	15.35	13.65
20:1	ND	ND	0.46
Sum MUFA	11.03	18.02	16.66
18:3n3	1.99	0.32	0.62
Sum (n-3)	1.99	0.32	0.62
18:2- cis n6	24.08	6.83	4.71
20:2n6	16.21	ND	0.15
20:3n6	ND	ND	0.38
Sum (n-6)	40.29	6.83	5.25
Sum PUFA	42.28	7.15	5.87
n-3/n-6	0.05	0.05	0.12

ND: not-detected.

#### Vitamins

Data on the four types of vitamins such as Vitamin A, B1, B2 and C in the BSFL are presented in Table 4. Vitamin A (Beta carotene) was not found in every BSFL while vitamins B1 and B2 were detected in OVN1 and OVN2 BSFL. Meanwhile, vitamin C occurred in all BSFL preparations.

**Table 4:** Concentration of vitamins in the (mg 100g^-1^).

Vitamins	SPR	OVN1	OVN2
Vitamin A (Beta carotene)	ND	ND	ND
Vitamin B1 (Thiamine)	ND	2.00	1.98
Vitamin B2 (Riboflavin)	ND	5.00	24.74
Vitamin C (Ascorbic acid)	0.37	0.22	0.19

ND: not detected.

#### Mineral and heavy metal composition

Table 5 shows the composition of minerals found in the three different types of BSFL. The highest amount of mineral noticed in BSFL was calcium (Ca), followed by potassium (K), phosphorus (P), sodium (Na), magnesium (Mg), zinc (Zn), iron (Fe), manganese (Mn) and copper (Cu). There were no appreciable differences in the concentrations of Ca and Na in every BSFL. This is unlike the mineral concentrations in the samples. Ca and Na concentrations were found to be highest in OVN1, followed by SPR and OVN2 while the other minerals such as K, P, Mg, Zn, Fe and Cu were most abundance in the SPR BSFL. With the exception of Zn, most of the mineral concentrations were found less abundant in the OVN2.

**Table 5:** Mineral composition of three different types of BSFL.

Minerals (mg kg^-1^)	BSFL		
	SPR	OVN1	OVN2
Ca	21176.00 ± 3611.61^ab^	26515.88 ± 4502.47^b^	13005.12 ± 595.94^a^
K	13156.34 ± 386.34^b^	11256.11 ± 335.60^a^	12847.07 ± 66.22^b^
P^1^	9100	7800	6000
Na	4084.13 ± 627.56^ab^	5028.42 ± 450.63^b^	3385.20 ± 110.93^a^
Mg	3616.55 ± 158.25^b^	3310.19 ± 224.71^b^	2505.98 ± 44.74^a^
Zn	1209.63 ± 84.50^b^	303.07 ± 20.80^a^	984.36 ± 199.44^b^
Fe	689.24 ± 37.39^b^	300.75 ± 5.00^a^	313.48 ± 60.45^a^
Mn	134.93 ± 1.10^a^	140.12 ± 1.79^a^	162.51 ± 2.30^b^
Cu	29.10 ± 5.85^b^	13.69 ± 0.40^a^	16.38 ± 0.44^a^

Results are means ± SE of analysis of three samples (n = 3).

^1^: Results are from 1 sample (n = 1).

Within a row, means with the same letters are not significantly different (P>0.05).

Table 6 shows the heavy metal composition observed in the BSFL. The Zn concentration was highest compared to the other heavy metals. Concentration of this mineral in SPR and OVN2 exceeded that in OVN1 while the other heavy metal concentrations were below 1000 mg kg^-1^. Most of the heavy metals were higher in SPR and lower in OVN1.

**Table 6:** Heavy metal composition of three different types of BSFL.

	BSFL		
Heavy metals (mg kg^-1^)	SPR	OVN1	OVN2
Zn	1209.63 ± 84.50^b^	303.07 ± 20.80^a^	984.36 ± 199.44^b^
Fe	689.24 ± 37.39^b^	300.75 ± 5.00^a^	313.48 ± 60.45^a^
Al	595.66 ± 133.24^b^	201.88 ± 38.30^a^	327.87 ± 5.45^ab^
Mn	134.93 ± 1.10^a^	140.12 ± 1.79^a^	162.51 ± 2.30^b^
Cr	40.88 ± 8.84^b^	9.08 ± 4.23^a^	11.55 ± 1.78^a^
Cu	29.10 ± 5.85^b^	13.69 ± 0.40^a^	16.38 ± 0.44^a^
Pb	15.36 ± 0.62^c^	3.94 ± 0.92^b^	0.70 ± 0.53^a^
Ni	14.10 ± 6.48^b^	ND^a^	2.60 ± 0.43^ab^
Bi	9.48 ± 2.75^a^	4.28 ± 0.99^a^	4.97 ± 0.35^a^
As	4.63 ± 1.52^a^	3.98 ± 2.16^a^	0.61 ± 0.31^a^
V	3.36 ± 0.10^b^	3.59 ± 0.03^b^	3.01 ± 0.07^a^
Co	2.28 ± 1.45^a^	ND^a^	ND^a^
Ga	2.07 ± 1.30^a^	2.07 ± 1.02^a^	0.27 ± 0.27^a^
Cd	1.67 ± 0.39^b^	0.73 ± 0.12^a^	0.59 ± 0.07^a^
Ag	0.36 ± 0.17^a^	0.36 ± 0.08^a^	0.42 ± 0.12^a^
In	0.55 ± 0.55^a^	ND^a^	ND^a^

Results are means ± SE of analysis of three samples (n = 3); ND = not detected.

Within a row, means with the same letters are not significantly different (P>0.05).

ND: not detected.

#### Nucleotide composition

Table 7 presents the data on analysis of nucleotides in the BSFL. The inosine-5’-monophosphate (IMP) and uridine-5’-monophosphate (UMP) were detected in all the BSFL preparations. However, the highest concentration was found in OVN2, followed by OVN1 and SPR. The other nucleotides such as guanosine-5’- monophosphate (GMP), adenosine-5’-monophosphate (AMP), xanthosine-5’-monophosphate (XMP) and cytidine-5’-monophosphate (CMP) were observed in either SPR and OVN 2 or both.

**Table 7:** Nucleotide concentrations of three different types of BSFL (% protein).

Nucleotides	SPR	OVN1	OVN2
IMP	0.23	0.28	0.37
UMP	0.12	0.18	0.25
GMP	0.06	ND	0.07
AMP	0.11	ND	ND
XMP	ND	ND	0.11
CMP	0.03	ND	0.11

ND: not detected.

## Discussion

The proximate composition of BSFL was significantly influenced by the processing method. Spray-drying method yielded higher protein content than the oven drying method. On the contrary, higher lipid level was observed when BSFL was processed using an oven drying method. Spray drying has been reported to be better than the oven drying method in terms of efficiency and quality effects as a result of shorter time taken to dry the feed via the former method [22, 23]. The crude protein contents of SPR and OVN1 were similar to the values reported by Kroeckel *et al*. [24], while the value for OVN2 was lower. Meanwhile, the lipid content of BSFL in the present study was higher than the lipid contents of fishmeal and BSFL as reported by Barroso *et al*. [25] where values ranged 15.6–18.0%, but it was similar to the lipid values observed by Newton *et al*. [26] and Sheppard *et al*. [27]. It was previously reported that the yield quantity and quality of BSFL protein depend on the stage of development of the insect and also the processing method [28]. The developmental stages of the BSF can be one of the factors that affect the lipid composition of the BSFL. High lipid composition can be observed in the pre-pupa stage of the insect due to the metabolic turnover that takes place during the process of metamorphosis [2, 29, 30]. In view of the knowledge gaps in this area and the complexities involved, it is difficult to establish actual mechanisms or to relate the pre-pupal stages to specific processes occurring at these stages of transformation.

Besides the developmental stages, feed consumed by the insects is another important factor that can influence the biochemical composition of the larvae [24]. In this study, the larvae from SPR and OVN1 were fed with organic, agro-industrial by-products while OVN2 were fed with kitchen and food waste. High lipid content of the OVN2 obviously resulted from the food waste that usually contains large amounts of fat. Hence, the use of food waste as a sole source of feed may not be the best substrate for insects that will be used as aquaculture feed since lipids are susceptible to degradation. The ash content of BSFL obtained in this study was less than that in BSFL as observed by Barroso *et al*. [25].

The fibre content is normally observed to identify the amount of chitin in the BSFL since this polysaccharide is the most common form of fibre in insects [6]. Highest fibre concentration was noticed in the SPR, followed by OVN1 and OVN2. The fibre content in insects depends on the stage of life cycle. As the larvae progress towards the pre-pupa stage and eventually the pupation stage, chitin content starts to increase [31]. Soetemans *et al*. [14] have noticed this phenomenon in BSF. Obviously, this will influence the digestibility. Fines and Holt [18] have emphasized the importance of optimizing the amount of chitin in the feed according to chitinolytic activity in the gut of a given fish and its ability to digest this substance in deciding the rate of inclusion [32].

In terms of amino acids, SPR had a slightly better profile compared to OVN1 and OVN2. Generally, the amino acid profile of the three BSFL was quite similar to fish meal [33] which was considered as the protein with the best amino acid profile for fish nutrition. The amino acid composition of BSFL in the present study was similar to that of BSFL in the study conducted by Cummins *et al*. [4]. Concordant results were reported by St-Hilaire *et al*. [34] using BSFL fed swine manure where leucine, lysine and valine were also amongst the highest in essential amino acids (EAA). The values of other essential amino acids obtained in this study were also comparable to the values presented by St-Hilaire *et al*. [34]. Generally, the amino acid profiles of insects belonging to the Order Diptera such as *H*. *illucens* and *Musca domestica*, are better than the soybean meal and can be used as a suitable replacement of fish meal in the aquaculture feed formulation compared to soybean meal [25].

The saturated fatty acid (SFA) concentrations found in the BSFL were higher compared to the unsaturated fatty acids. Among the three BSFL, the SPR was found to contain the lowest SFA. This is expected due to the different feeds supplied and the processing method applied that influenced the fatty acid profile. Lauric acid and palmitic acid concentrations were appreciably higher compared to other SFA. Similarly, St-Hilaire *et al*. [34] and Sealey *et al*. [35] also reported high levels of lauric acid and palmitic acids in BSFL. Thompson [36] reported that there are only some insects that naturally contain long-chain unsaturated fatty acids. BSFL and feed containing BSFL usually have low concentration of unsaturated fatty acids [34].

Insects such as black soldier fly can be an interesting candidate in terms of nutritional contents of minerals (K, Na, Ca, Cu, Fe, Zn, Mn, and P) [37]. Vitamin A was not detected in the tested BSFL. Some insects are not the best source of vitamin A [6]. Vitamins are needed in small quantities in most animal feeds. They assist in maintenance of normal metabolic and physiological functions [38]. Both B1 (thiamine) and B2 (riboflavin) vitamins were found in OVN 1 and OVN2 BSFL samples. Other insect species are also known to contain these B-complex vitamins. A study by Bukkens [39] presented several species of insects where thiamine ranged from 0.1 mg to 4 mg per 100 g of dry matter. This is an essential vitamin that acts as a co-enzyme to metabolize carbohydrate to produce energy. Riboflavin, a yellow-coloured pigment, is present in many insects in an extremely variable quantity. For example, Finke [40] reported that mealworm contained about 8.1 mg/kg riboflavin as compared to 5–24.74 mg/100g in the present study. Vitamin C was found in all BSFL samples (0.19–0.37 mg 100g^-1^). Presence of this vitamin in the BSFL is important to support the normal growth performance and feed utilisation of the farmed animals [41].

It has been reported that mineral composition of fly larvae is strongly influenced by the type of processing methods [19] and variation in the diets used [42]. In the present study, OVN1 BSFL was observed to contain higher concentration of minerals and lower amounts of heavy metals than SPR and OVN2. Kouřimská and Adámková [37] stated that the BSFL may contain heavy metals due to their uptake from the environment. Suitable food substrate should be considered in order to obtain desirable nutritional content of BSFL. Apart from that, for achieving a safe insect meal for use as a dietary feed ingredient, setting up of sanitation procedures for use of bio-waste and managing diseases, pesticides and heavy metals should be given due attention [43].

Insects such as *H*. *illucens* are also a good source of nucleotides. Nucleotides are natural biochemical substances that consist of a purine or pyrimidine base. Nucleotides can be obtained from a feed that is high in protein [44]. These are among the taste substances that have been commonly used as feeding stimulant in animal feeds [45, 46]. To the best of our knowledge, this is the first report that presents the concentration of nucleotides in the BSFL as feed ingredients are not routinely analyzed for their concentration of nucleotides. Based on these results, the concentration of most nucleotides found in this study are much higher than the concentration of nucleotides such as CMP, AMP, GMP, UMP and IMP of several feed ingredients including the fish meal where the amounts range from 0.001–0.0035 mg/g [47]. The quantitative differences noticed in the nucleotide components were evident but how these differences contribute to the total nucleotide content of the feeds remains unclear. While no information on this aspect has been published on BSFL, Kay and Vrede [48] have presented a general assessment of the nucleotide profile in animals that linked the variations to the nitrogenous base. In the specific context of fish, the significance of nucleotides has been established in several earlier studies, especially in cellular metabolism [49], as carriers of chemical energy in cells and enzyme cofactors [48], oxidative stress mitigation [50], disease resistance [51] and growth stimulation [52]. Shiau *et al*. [53] have highlighted the significant role of nucleotides in developing, maintaining, and enhancing the immune system of fish. Data obtained in this study contributes to filling the knowledge gaps as well as proposing the appropriate method of processing BSFL for best results on the farmed fish.

There is a growing number of publications highlighting the importance of the nutrient profiles in BSFL. Józefiak *et al*. [54] observed their effect on gut microbiome composition and intestinal health of sturgeon. Tippayadara *et al*. [55] documented positive influence on feed efficiency, haematological parameters, and somatic indices in tilapia. Concordant views have been expressed earlier by other authors [56–60].

Thus, BSFL meal, especially the OVN2 BSFL, that contains a high concentration of nucleotides than the fish meal can be recommended as one of the high potential protein sources in animal feeds.

## Conclusions

It can be concluded that BSFL has a good nutritional profile in terms of proximate composition, amino acids, fatty acids, vitamins, minerals and nucleotides. However, the high lipid concentration influenced by the substrate will limit the use of BSFL in the diet formulation. Methods of feed processing affect the nutritional value of the insect-based aquaculture feed. To improve the omega-3 fatty acids in the BSFL, supplementing the feed with high levels of these nutrients from sources such as fish offal can be considered. Nevertheless, a better option is to explore the potential of BSFL in converting food waste into high-quality protein. This will necessitate investigations on determining the appropriate growth substrates that strongly influence the nutritional value of insect meal to support protein enrichment of BSFL with a balanced amino acid profile and managing the lipid range. The versatility and ability of BSFL to change its amino acid and fatty acid composition in response to growth media offers a significant advantage of replacing fish meal from aquafeeds. Further research is needed on quality and quantity of BSFL inclusion and digestibility of the fish to enhance the contribution of the insect meal in making the aquafeed sector more sustainable.

Presence of B-complex vitamins and several minerals that are critically important for growth and metabolism of fed species substantiates the argument for increasing the proportion of BSFL in aquaculture nutrition. Amounts of these substances can be modulated by changing the insect growth medium during rearing and selecting the feed production methods through follow-up investigations. Significant quantities of nucleotides in BSFL should change the outlook towards these nutrients that are largely ignored in feed formulation despite their being the building blocks of nucleic acids. Due to the fact fish cannot synthesise the amounts of nucleotides that the body needs, these can be supplemented through the diet comprising a significant portion of BSFL.

This study should motivate more interest in future research for better understanding the role of nucleotides in metabolism, feed efficiency, protein biosynthesis and growth, and enhancing the stamina and resilience of the cultured species.

## Supporting information

S1 File Raw data Table 1. (PDF)

S2 File Raw data Table 5. (PDF)

S3 File Raw data Table 6. (PDF)

## References

[1] Costello C, Cao L, Gelcich S, Cisneros-Mata M, et al. The future of food from the sea. Nature. 2020;588:95–100. doi: 10.1038/s41586-020-2616-y 32814903

[2] English G, Wanger G, Colombo SM. A review of advancements in black soldier fly (Hermetia illucens) production for dietary inclusion in salmonid feeds. Journal of Agriculture and Food Research. 2021;5:100164.

[3] Bondari K, Sheppard D. Soldier fly as feed in commercial fish production. Aquaculture. 1981;24:103–109.

[4] Cummins VC, Rawles SD, Thompson KR, Velasquez A, Kobayashi Y, Hager J, et al. Evaluation of black soldier fly (Hermetia ilucens) larvae meal as partial or total replacement of marine fish meal in practical diets for Pacific white shrimp (Litopenaeus vannamei). Aquaculture. 2017; 743:337–344.

[5] Sheppard DC, Newton LG, Thompson SA, Savage S. A value added manure management systems using the black soldier fly. Bioresource Technology. 1994;50(3):275–279.

[6] van Huis A, Itterbeeck J, Klunder H, Mertens E, Halloran A, Muir G, et al. Edible insects: future prospects for food and feed security. FAO Forestry paper;2013 No 171, 187p.

[7] Oonincx DGAB, van Itterbeeck J, Heetkamp MJW, van den Brand H, van Loon JJA, van Huis A. An Exploration on greenhouse gas and ammonia production by insect species suitable for animal or human consumption. PLoS ONE. 2010;5(12): e14445. doi: 10.1371/journal.pone.0014445 21206900 PMC3012052

[8] Wang YS, Shelomi M. Review of Black soldier fly (Hermetia illucens) as animal feed and human food. Foods. 2017;6:91. doi: 10.3390/foods6100091 29057841 PMC5664030

[9] Alvarez D, Wilkinson KA, Treilho M, Tene N, Castillo D, Sauvian M, et al. P. Prospecting peptides isolated from black soldier fly (Diptera: Stratiomydae) with antimicrobial activity against Helicobacter pylori (Campylobacterales: Helicobacteraceae). Journal of Insect Science. 2019;19 (6):17. doi: 10.1093/jisesa/iez120 31865367 PMC6925832

[10] Manniello MD, Moretta A, Salvia R, Scieuzo C, Lucchetti D, Vogel H, et al. Insect antimicrobial peptides: potential weapons to counteract the antibiotic resistance. Cellular and Molecular Life Sciences.2021; 78:1–24. doi: 10.1007/s00018-020-03576-x 33595669 PMC8164593

[11] Moretta A, Scieuzo C, Petrone AM, Salvia R, Manniello MD, Franco A, et al. Antimicrobial peptides: A new hope in biomedical and pharmaceutical fields. Frontiers in Cellular and Infection Microbiology. 2021;11:668632. doi: 10.3389/fcimb.2021.668632 34195099 PMC8238046

[12] Xia J, Ge C, Yao H. Antimicrobial peptides from black soldier fly (Hermetia illucens) as potential antimicrobial factors representing an alternative to antibiotics in livestock farming. Animals. 2021;11,1937. doi: 10.3390/ani11071937 34209689 PMC8300228

[13] Hahn T, Tafi E, Paul A, Salvia R, Falabella P, Zibek S. Current state of chitin purification and chitosan production from insects. Journal of Chemical Technology and Biotechnology. 2020; 95:2775–2795.

[14] Soetemans L, Uyttebroek M, Bastiaens L. Characteristics of chitin extracted from black soldier fly in different life stages. International Journal of Biological Macromolecules. 2020;165:3206–3214. doi: 10.1016/j.ijbiomac.2020.11.041 33181213

[15] Triunfo M, Tafi E, Guarnieri A, Scieuzo C, Hahn T, Zibek S, et al. Insect chitin-based nanomaterials for innovative cosmetics and cosmeceuticals. Cosmetics. 2021; 8:40.

[16] Franco A, Scieuzo C, Salvia R, Petrone AM, Tafi E, et al. Lipids from Hermetia illucens, an Innovative and Sustainable Source. Sustainability. 2021; 13 (18): 10198.

[17] Abd -ElHack ME, Shafi M., Alghamdi WY, Abdelnour S, Shehata AM, Noreldin AE, et al. Black soldier fly (Hermetia illucens) meal as a promising feed ingredient for poultry: A comprehensive review. Agriculture. 2020;10:339.

[18] Bessa LW, Pieterse E, Marais J, Hoffman LC. Why for feed and not for human consumption? The black soldier fly larvae. Comprehensive Reviews in Food Science and Food Safety. 2020;19:2747–2763. doi: 10.1111/1541-4337.12609 33336973

[19] Fasakin EA, Balogun AM, Ajayi OO. Evaluation of full-fat and defatted maggot meals in the feeding of clariid catfish *Clarias gariepinus* fingerlings. Aquaculture Research. 2003;34:733–738.

[20] Spranghers T, Ottoboni M, Klootwijk C, Ovyn A, Deboosere S., et al. Nutritional composition of black soldier fly (Hermetia illucens) prepupae reared on different organic waste substrates: nutritional composition of black soldier fly. Journal of the Science of Food and Agriculture. 2017;97(8):2594–2600. doi: 10.1002/jsfa.8081 27734508

[21] AOAC (Association of Official Analytical Chemists). In: Cunniff P, editor. Official methods of analysis of AOAC international. Volume 1, 5th revision, 16th ed. Washington, Association of Office Analytical Chemists International; 1999.

[22] Guin é RPF. The drying of foods and its effect on the physical-chemical, sensorial and nutritional properties. International Journal of Food Engineering. 2018;4(2):93–100.

[23] Raza N, Arshad MU, Anjum FM, Saeed F, Maan AA, Ul Ain H.B. Impact of drying methods on composition and functional properties of date powder procured from different cultivars. Food Science & Nutrition. 2019;7:2345–2352. doi: 10.1002/fsn3.1081 31367363 PMC6657709

[24] Kroeckel S, Harjes AGE, Roth I, Katz H, Wuertz S, Susenbeth A, et al. When a turbot catches a fly: Evaluation of a pre-pupae meal of the Black soldier fly (Hermetia ilucens) as fish meal substitute. Aquaculture. 2012;364–365:345–352.

[25] Barroso F, Haro C, Sánchez-Muros M, Venegas E, Martínez-Sánchez A, Pérez-Bañón C. The potential of various insect species for use as food for fish. Aquaculture. 2014;422–423:193–201.

[26] Newton GL, Booram CV, Barker RW, Hale OM. Dried *Hermetia illucens* larvae meal as a supplement for pig. Journal of Animal Science. 1977;44(3):395–400.

[27] Sheppard DC, Tomberlin JK, Joyce JA, Kiser BC, Sumner SM. Rearing methods for the black soldier fly (Diptera: Stratiomyidae). Journal of Medical Entomology, 2002;39(4):695–698. doi: 10.1603/0022-2585-39.4.695 12144307

[28] Aniebo AO, Owen OJ. Effects of age and method of drying on the proximate composition of housefly larvae (Musca domestica Linnaeus) meal (HFLM). Pakistan Journal of Nutrition. 2010; 9:485–487.

[29] Magalhães R, Sánchez-López A, Leal RS, Martínez-Llorens S, Oliva-Teles A, Peres H. Black soldier fly (Hermetia illucens) pre-pupae meal as a fish meal replacement in diets for European seabass (Dicentrarchus labrax). Aquaculture. 2017;476:79–85.

[30] Panini RL, Freitas LEL, Guimarães AM, Rios C, da-Silva MFO, Viera FN, et al. Potential use of mealworms as an alternative protein source for Pacific white shrimp: Digestibility and performance. Aquaculture. 2017;473:115–120.

[31] Kramer KJ, Koga D. Insect chitin. Insect Biochem. 1986;16(6):851–877.

[32] Fines BC, Holt GJ. Chitinase and apparent digestibility of chitin in the digestive tract of juvenile cobia, *Rachycentron canadum*. Aquaculture. 2010;303:34–39.

[33] Henry M, Gasco L, Piccolo G, Fountoulaki E. Review on the use of insects in the diets of farmed fish: past and future. Animal Feed Science Technology. 2015;203:1–22.

[34] St-Hilaire S, Cranfill K, McGuire M, Mosley E, Tomberlin J, Newton L, et al. Fish offal recycling by the black soldier fly produces a foodstuff high in omega-3 fatty acids. Journal of the World Aquaculture Society. 2007; 38:309–313.

[35] Sealey WM, Gaylord TG, Barrows FT, Tomberlin JK, McGuire MA, Ross C, et al. Sensory analysis of rainbow trout, (Oncorhynchus mykiss), fed enriched black soldier fly (Hermetia illucens) prepupae. Journal of the World Aquaculture Society. 2011;42(1):34–45.

[36] Thompson SN A review and comparative characterization of the fatty acid composition of seven insect orders. Comparative Biochemistry and Physiology. 1973;45B:467–482.

[37] Kouřimská L, Adámková A. Nutritional and sensory quality of edible insects. NFS Journal. 2016;4:22–26.

[38] Pillay TVR, Kutty MN. Aquaculture: Principles and Practices. Oxford, UK: Blackwell Publishing Ltd.; 2005.

[39] Bukkens SGF. Insects in the human diet: nutritional aspects. In. Paoletti MG, editor. Ecological implications of mini-livestock; role of rodents, frogs, snails, and insects for sustainable development. New Hampshire: Science Publishers; 2005. p. 545–577.

[40] Finke MD. Complete nutrient composition of commercially raised invertebrates used as food for insectivores. Zoo Biology. 2002;21(3):269–285.

[41] Adewolu MA, Aro OO. Growth, feed utilization and haematology of (Clarias gariepinus Burchell, 1822) fingerlings fed diets containing different levels of vitamin C. The American Journal of Applied Science. 2009;6(9):1675–1681.

[42] Newton GL, Sheppard D, Watson W, Burtle GJ, Dove RC, Tomberlin JK, et al. The black soldier fly, (Hermetia illucens) as a manure management/resource recovery tool. State of the Science, Animal Manure and Waste Management, San Antonio: TX. 2005 Jan 5–7.

[43] Makkar HPS, Tran G, Heuzé V, Ankers P. State-of-the-art on use of insects as animal feed. Animal Feed Science and Technology. 2014;197:1–33.

[44] Carver JD, Walker WA. The role of nucleotides in human nutrition. The Journal of Nutritional Biochemistry. 1995;6(2):58–72.

[45] Kasumyan AO, Døving KB. Taste preferences in fish. Fish and Fisheries. 2003; 4:289–347.

[46] Li P, Gatlin DM III. Nucleotide nutrition in fish: current knowledge and future applications. Aquaculture. 2006;251:141–152.

[47] Mateo C, Stein, H. Nucleotides may have a role in nutrition of young pigs. Alltech’s 20th Annual Symposium. 2004. p. 1–21.

[48] Kay AD, Vrede T. Nucleotides. In: Encyclopaedia of Ecology, Evolutionary and Biochemical Aspects. London: Academic Press: 2008. P. 1472–1481.

[49] Do-Huu H. Overview of the application of nucleotide in aquaculture. Journal of Coastal Life Medicine. 2016;4:816–823.

[50] Hossain NS, Koshio S, Ishikawa M, et al. Dietary nucleotide administration influences growth, immune responses and oxidative stress resistance of juvenile red sea bream (Pagrus major). Aquaculture. 2016;355:41–49.

[51] Burrells C, Williams PD, Forna PF. Dietary nucleotides: a novel supplement in fish feeds.1. Effects on resistance to disease in salmonids. Aquaculture. 2001; 199:159–169.

[52] Tahmasebi-Kohyani A., Keyvanshokooh S., Nematollahi A., Mahmoudi N., Pasha-Zanooshi H. Dietary administration of nucleotides to enhance growth, humoral immune responses and disease resistance of the rainbow trout (Oncorhynchus mykiss) fingerlings. Fish and Shellfish Immunology. 2011; 30:189–193. doi: 10.1016/j.fsi.2010.10.005 20955799

[53] Shiau SY, Gabaudan J, Lin YH. Dietary nucleotide supplementation enhances immune responses and survival to *Streptococcus iniae* in hybrid tilapia fed diet containing low fish meal. Aquaculture. 2015;2:77–81.

[54] Józefiak A., Nogales-Mérida S, Rawski M., et al. Effects of insect diets on the gastrointestinal tract health and growth performance of Siberian sturgeon (Acipenser baerii Brandt, 1869). BMC Veterinary Research. 2019;15:348. doi: 10.1186/s12917-019-2070-y 31623627 PMC6798509

[55] Tippayadara N., Dawood MAO, Krutmuang P, Hoseinifar SH, et al. (2021). Replacement of Fish Meal by Black Soldier Fly (Hermetia illucens) Larvae Meal: Effects on Growth, Haematology, and Skin Mucus Immunity of Nile Tilapia, Oreochromis niloticus. Animals. 2021;11(1):193. doi: 10.3390/ani11010193 33467482 PMC7830215

[56] Rana KS, Salam MA, Hashem S, Islam MA. Development of black soldier fly larvae production technique as an alternate fish feed. International Journal of Research in Fisheries and Aquaculture. 2015;5(1):41–47.

[57] Shumo M., Osuga I.M., Khamis F.M., Tanga C.M., Fiaboe K.K.M., Subramanian S., et al. The nutritive value of black soldier fly larvae reared on common organic waste streams in Kenya. Scientific Reports. 2019;9:1–13. doi: 10.1038/s41598-018-37186-2 31300713 PMC6626136

[58] Wang G., Peng K., Hu J., Yi C., Chen X., Wu H., et al. Evaluation of defatted black soldier fly (Hermetia illucens L.) larvae meal as an alternative protein ingredient for juvenile Japanese seabass (Lateolabrax japonicus) diets. Aquaculture. 2019;507:144–154.

[59] Abdel-Tawwab M, Khalil RH, Metwally AA, Shakweer MS, Khallaf MA, Abdel-Latif HM. Effects of black soldier fly (Hermetia illucens L.) larvae meal on growth performance, organs-somatic indices, body composition, and hemato-biochemical variables of European sea bass, *Dicentrarchus labrax*. Aquaculture. 2020;522: 735136.

[60] Fisher H, Collins SA, Hanson C, Mason B, Colombo S, Anderson D. Black soldier fly larvae meal as a protein source in low fish meal diets for Atlantic salmon (Salmo salar). Aquaculture. 2020;521:734978.

