# Supplementary material for: Nutritional value of black soldier fly (Hermetia illucens) larvae processed by different methods
Source: PLoS One. 2022 Feb 25;17(2):e0263924. doi: 10.1371/journal.pone.0263924 (PMC8880436; doi:10.1371/journal.pone.0263924)
Supplement: S1 File — (PDF) [file pone.0263924.s001.pdf]

Raw data for Table 1. Proximate composition of the BSFL (% DM)

	Protein	Lipid	Fibre	Moisture	Ash	NFE
SPR1	48.29	25.45	11.08	7.21	8.34	6.84
SPR2	48.20	25.79	10.14	7.06	8.34	7.53
SPR3	48.10	25.83	8.67	7.04	8.13	9.26
Average	48.20	25.69	9.96	7.10	8.27	7.88
SD	0.09	0.21	1.21	0.09	0.12	1.24
SE	0.05	0.12	0.70	0.05	0.07	0.72

	Protein	Lipid	Fibre	Moisture	Ash	NFE
OVN1.1	47.30	28.52	9.55	3.26	8.21	6.42
OVN1.2	47.73	28.35	9.50	3.21	8.10	6.32
OVN1.3	47.33	28.40	9.41	3.15	8.25	6.61
Average	47.45	28.43	9.48	3.21	8.19	6.45
SD	0.24	0.09	0.07	0.06	0.08	0.15
SE	0.14	0.05	0.04	0.03	0.05	0.09

	Protein	Lipid	Fibre	Moisture	Ash	NFE
OVN2.1	39.40	37.98	7.39	5.31	7.29	7.94
OVN2.2	39.65	38.54	7.48	5.37	7.21	7.12
OVN2.3	39.08	38.56	7.37	5.28	7.28	7.71
Average	39.38	38.36	7.41	5.32	7.26	7.59
SD	0.29	0.33	0.05	0.05	0.04	0.42
SE	0.16	0.19	0.03	0.03	0.03	0.24
