# Supplementary material for: Nutritional value of black soldier fly (Hermetia illucens) larvae processed by different methods
Source: PLoS One. 2022 Feb 25;17(2):e0263924. doi: 10.1371/journal.pone.0263924 (PMC8880436; doi:10.1371/journal.pone.0263924)
Supplement: S2 File — (PDF) [file pone.0263924.s002.pdf]

Raw data for Table 5. Mineral composition of three different types of BSFL

	Ca	K	Na	Mg	Zn	Fe	Mn	Cu
SPR 1	18071.50	13868.24	5240.31	3591.80	1086.87	756.37	137.09	40.51
SPR 2	28376.51	13060.51	3929.06	3902.18	1170.41	684.21	133.51	25.66
SPR 3	17079.99	12540.26	3083.03	3355.68	1371.61	627.15	134.18	21.14
Average	21176.00	13156.34	4084.13	3616.55	1209.63	689.24	134.93	29.10
SD	6255.50	669.16	1086.97	274.09	146.36	64.75	1.90	10.13
SE	3611.62	386.34	627.56	158.25	84.50	37.39	1.10	5.85

	Ca	K	Na	Mg	Zn	Fe	Mn	Cu
OVN1.1	31714.04	11923.69	5371.42	3579.31	339.96	293.37	141.28	14.04
OVN1.2	17548.83	10862.01	5578.69	2863.91	267.99	310.29	142.46	14.15
OVN1.3	30284.78	10982.63	4135.13	3487.35	301.26	298.60	136.60	12.90
Average	26515.88	11256.11	5028.42	3310.19	303.07	300.75	140.12	13.69
SD	7798.51	581.28	780.52	389.22	36.02	8.67	3.10	0.69
SE	4502.47	335.60	450.63	224.71	20.80	5.00	1.79	0.40

	Ca	K	Na	Mg	Zn	Fe	Mn	Cu
OVN2.1	13528.18	12788.14	3596.80	2583.37	1370.38	275.85	162.39	17.05
OVN2.2	11816.10	12979.26	3337.17	2428.38	878.34	431.79	158.59	16.55
OVN2.3	13671.07	12773.80	3221.62	2506.20	704.35	232.79	166.57	15.54
Average	13005.12	12847.07	3385.20	2505.98	984.36	313.48	162.51	16.38
SD	1032.19	114.70	192.14	77.49	345.44	104.70	3.99	0.77
SE	595.94	66.22	110.93	44.74	199.44	60.45	2.30	0.44
