# Supplementary material for: Nutritional value of black soldier fly (Hermetia illucens) larvae processed by different methods
Source: PLoS One. 2022 Feb 25;17(2):e0263924. doi: 10.1371/journal.pone.0263924 (PMC8880436; doi:10.1371/journal.pone.0263924)
Supplement: S3 File — (PDF) [file pone.0263924.s003.pdf]

Raw data for Table 6. Heavy metal composition of three different types of BSFL

	SPR 1	SPR 2	SPR 3	Average	SD	SE
Zn	1086.87	1170.41	1371.61	1209.63	146.36	84.50
Fe	756.37	684.21	627.15	689.24	64.75	37.39
Al	340.22	657.64	789.12	595.66	230.78	133.24
Mn	137.09	133.51	134.18	134.93	1.90	1.10
Cr	55.29	42.56	24.79	40.88	15.32	8.84
Cu	40.51	25.66	21.14	29.10	10.13	5.85
Pb	16.57	14.51	14.98	15.36	1.08	0.62
Ni	26.62	10.78	4.90	14.10	11.23	6.48
Bi	14.96	7.16	6.32	9.48	4.77	2.75
As	3.20	3.02	7.67	4.63	2.63	1.52
V	3.36	3.53	3.18	3.36	0.18	0.10
Co	5.13	1.30	0.41	2.28	2.51	1.45
Ga	1.73	4.47	0.00	2.07	2.25	1.30
Cd	2.39	1.60	1.04	1.67	0.68	0.39
Ag	0.06	0.36	0.66	0.36	0.30	0.17
In	0.00	0.00	1.64	0.55	0.95	0.55

	OVN1.1	OVN1.2	OVN1.3	Average	SD	SE
Zn	339.96	267.99	301.26	303.07	36.02	20.80
Fe	293.37	310.29	298.60	300.75	8.67	5.00
Al	190.25	142.13	273.27	201.88	66.34	38.30
Mn	141.28	142.46	136.60	140.12	3.10	1.79
Cr	7.54	2.65	17.05	9.08	7.32	4.23
Cu	14.04	14.15	12.90	13.69	0.69	0.40
Pb	3.38	2.68	5.74	3.94	1.60	0.92
Ni	0.00	0.00	0.00	0.00	0.00	0.00
Bi	5.52	5.00	2.31	4.28	1.72	0.99
As	4.53	7.42	0.00	3.99	3.74	2.16
V	3.65	3.54	3.57	3.59	0.06	0.03
Co	0.00	0.00	0.00	0.00	0.00	0.00
Ga	2.22	3.75	0.24	2.07	1.76	1.02
Cd	0.85	0.84	0.49	0.73	0.21	0.12
Ag	0.21	0.46	0.41	0.36	0.13	0.08
In	0.00	0.00	0.00	0.00	0.00	0.00

	OVN2.1	OVN2.2	OVN2.3	Average	SD	SE
Zn	1370.38	878.34	704.35	984.36	345.44	199.44
Fe	275.85	431.79	232.79	313.48	104.70	60.45
Al	337.44	327.60	318.56	327.87	9.44	5.45
Mn	162.39	158.59	166.57	162.51	3.99	2.30
Cr	13.15	7.99	13.50	11.55	3.09	1.78
Cu	17.05	16.55	15.54	16.38	0.77	0.44
Pb	1.75	0.00	0.37	0.70	0.92	0.53
Ni	1.91	2.50	3.38	2.60	0.74	0.43
Bi	5.11	4.31	5.48	4.97	0.60	0.35
As	0.86	0.00	0.98	0.62	0.54	0.31
V	2.87	3.09	3.06	3.01	0.12	0.07
Co	0.00	0.00	0.00	0.00	0.00	0.00
Ga	0.82	0.00	0.00	0.27	0.47	0.27
Cd	0.67	0.64	0.45	0.59	0.12	0.07
Ag	0.61	0.19	0.45	0.42	0.21	0.12
In	0.00	0.00	0.00	0.00	0.00	0.00
